# Supplementary material for: Genome-Wide Characterization and Expression Profiling of HD-Zip Genes in ABA-Mediated Processes in Fragaria vesca
Source: Plants (Basel). 2022 Dec 4;11(23):3367. doi: 10.3390/plants11233367 (PMC9737017; doi:10.3390/plants11233367)
Supplement: Supplementary file 1 [file plants-11-03367-s001.zip › supplementary figures.pdf]

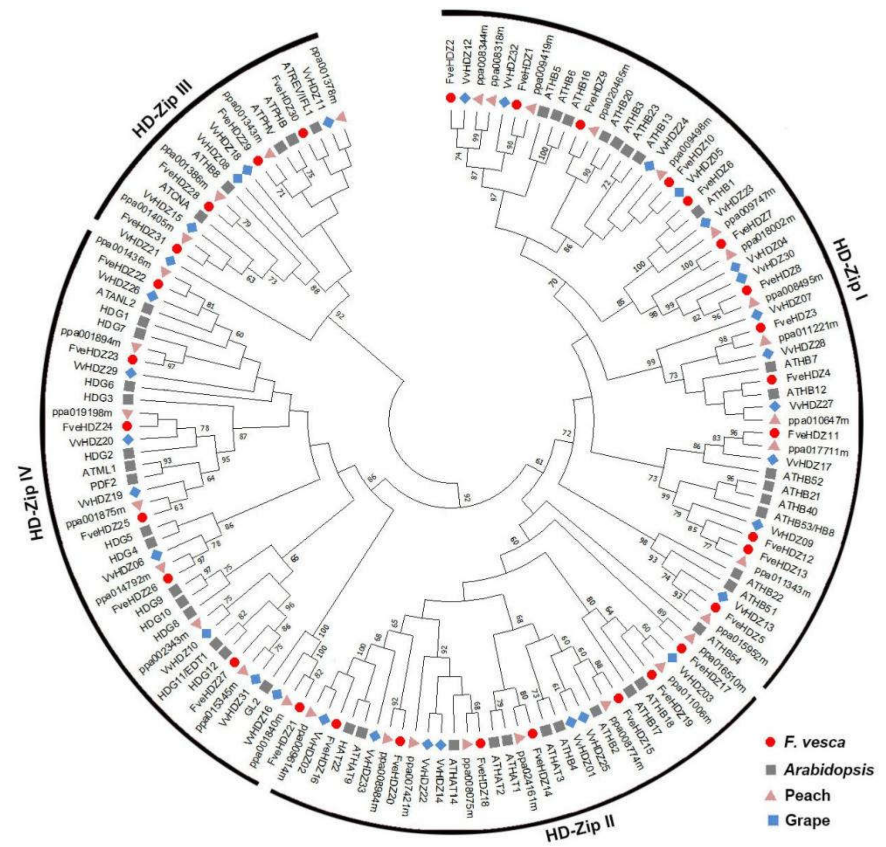

Figure S1. Phylogenetic clustering of HD-Zip proteins from *F. vesca*, *Arabidopsis*, grape, and peach. All of these HD-Zip proteins are well separated into four major subfamilies (I-IV). This unrooted phylogenetic tree was constructed by the Neighbor-Joining (NJ) method with 1500 bootstrap replicates in MEGA10. Bootstrap values > 60 are shown.

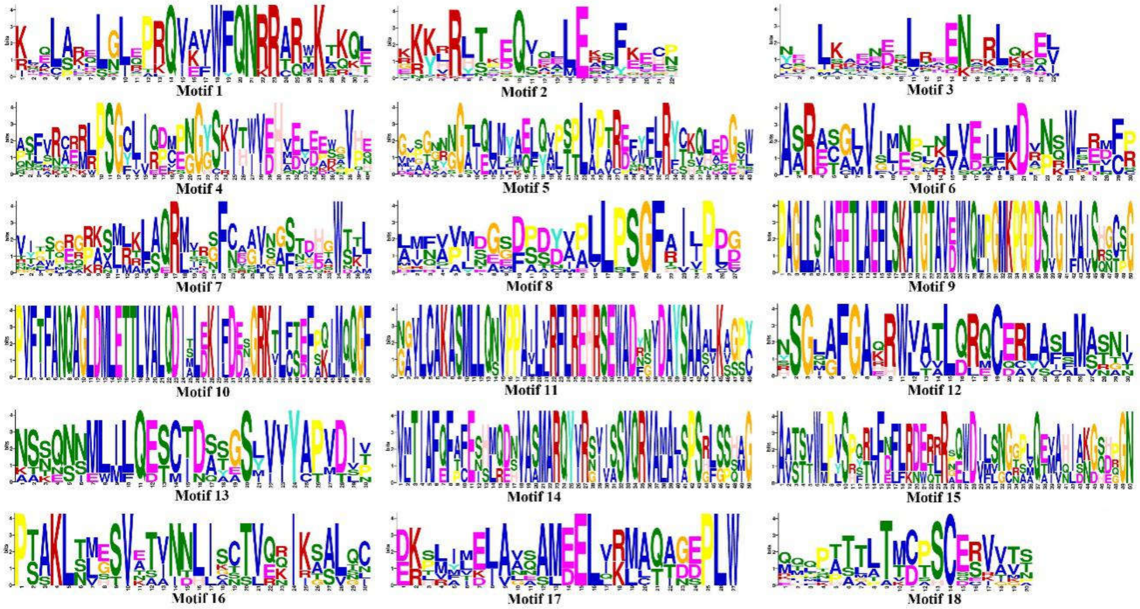

Figure S2. The sequence logos of each motif for *FveHDZ* proteins

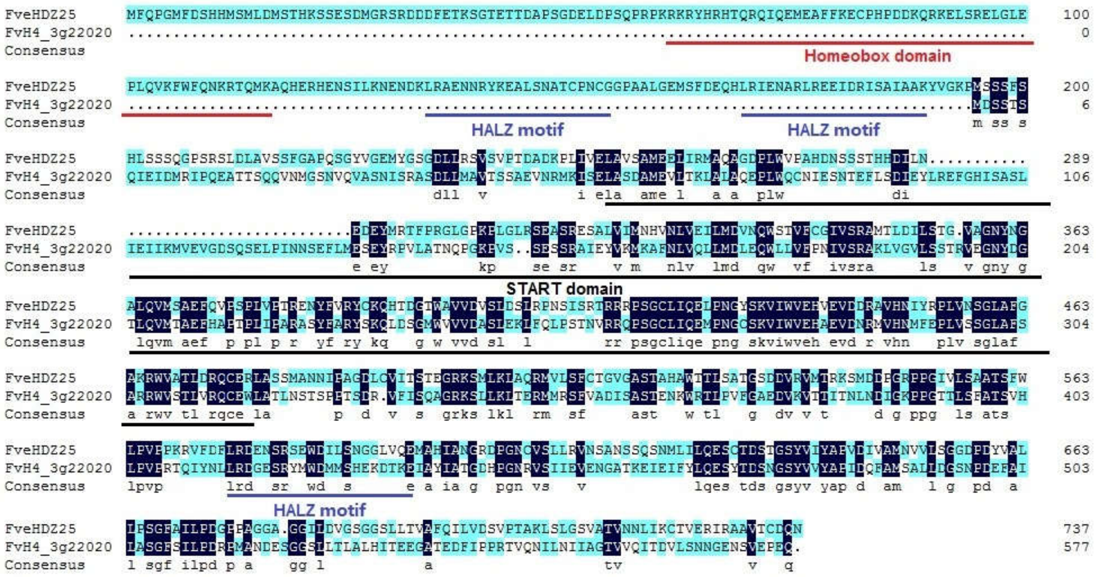

Figure S3. The protein sequence alignment of *FveHDZ25* and *FvH4_3g22020*. The core domain regions of these 2 proteins were marked by colored lines.

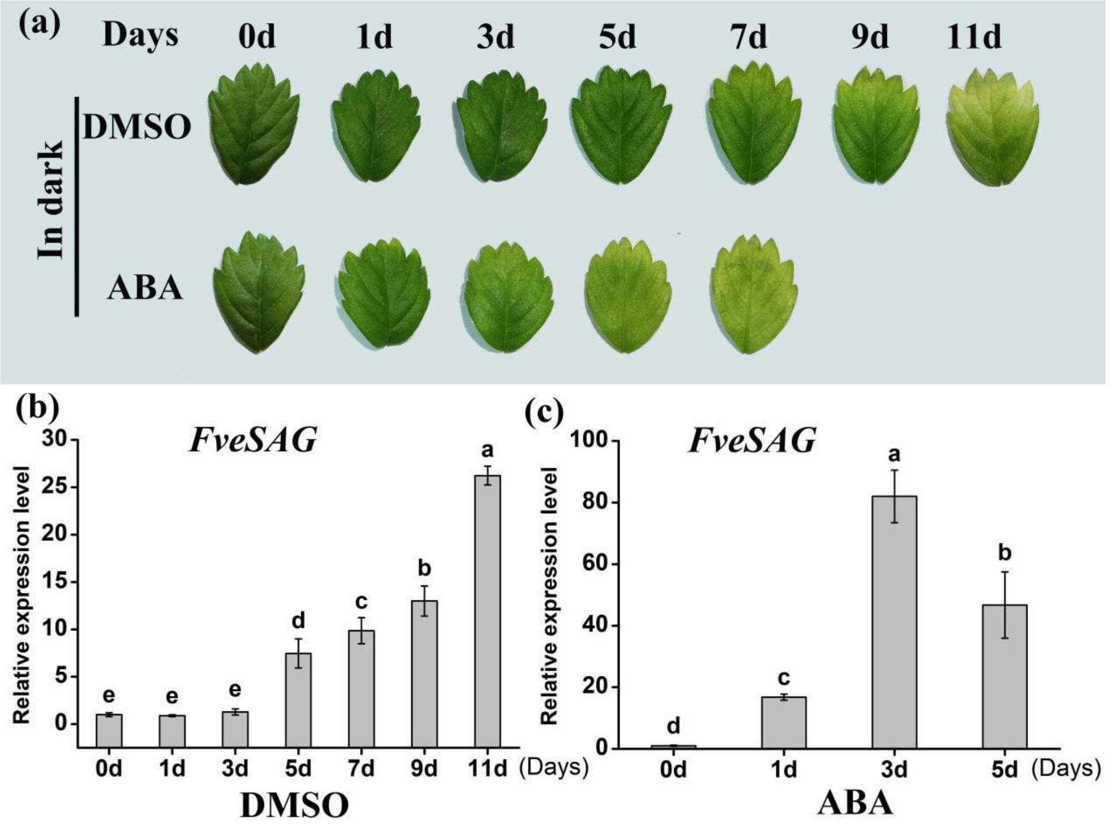

Figure S4. *F. vesca* leaves with different treatment days (**a**) and relative expression of the senescence-associated gene (*FveSAG*, FvH4_5g06770) in leaf by dark treatment with DMSO (**b**) or ABA (**c**). DMSO was the solvent of ABA. The expression of *FveSAG* at 0d was used as control and was normalized as "1". Data was shown as mean ± standard deviation derived from three biological replicates. The values with different letters were significant based on Duncan's multiple range tests ($P < 0.05$, $n = 3$).

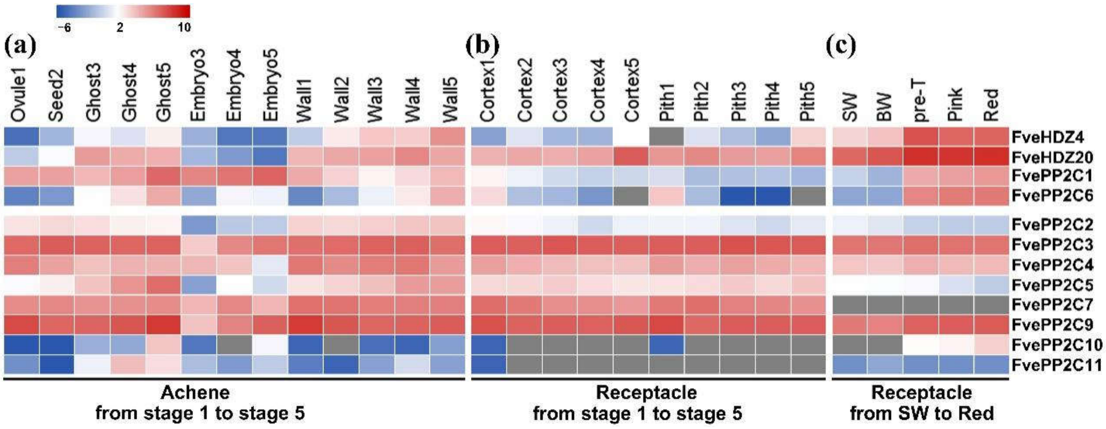

Figure S5. Expression profiles of the 10 *FvePP2Cs* and 2 *FveHDZs* in achene development (**a**) and receptacle development (**b**) and ripening (**c**). (**a**,**b**) Achenes included the ovule, seed, embryo, ghost and wall tissues, and receptacles included the cortex and pith tissues. Numbers after the tissues indicated different fruit development stages. Data from Kang et al. [43] and Hollender et al. [44]. (**c**) SW, small white; BW, big white; and Pre-T, pre-turning. Data from Gu et al. [45]. The heat map showed log2 "relative RPKM values" of individual *FveHDZ* genes. Gray boxes indicated undetectable expression of the genes.

>FvePP2C6-F1 (158bp)
GATGGATCTGTAAACCCTAATCTCTTAATTAAATGACTAATGTAAGATTTTTTGCGGTCC
ATTTTATCAAACATAAACCTTCACCAACTCAACCACAATCATTGAAAACTTGCCTAGTTT
ACAAATTTATACTCCAAAGCTTCCAAAGAAGAGAAGAG

>FvePP2C6-F2 (425bp)
CAGGAATTCTCTACGAGAGTAATATATTATGCGAGACATAATATGTTTGTATGCATATTA
CCCCCCTCTATTTCTACCTTATACAAATGTGTCTGTACGATTTTTTTTTTAAGTGAGAAA
TATAACACAACCTCACGAGTTAAAGTGCATTCAACCATCAATCACTTCAATGTTTTGCT
AAGTTATTACCCTTTGTCATAAATTCGATAATTACTTGCTTATATTGGATATACTAATGAT
TTACGATGATCTTTACCACTACCTCTTATAATTGAAAGATTAATATAACAATCTTGACTTA
ACGACTCGAGCAAATAATGTATTACTATTGTATTTCATTATTACTTGTCTTTTTGAGTAAA
TACTTAATTTCATAAATTTATTTATAGCCATAAGAGGCGTACATCAAATGTTGTCTTGAA
CCT

>FvePP2C1-F1 (298bp)
CAGTCTCATCAAATGCATGATAAAATGTGTACATTCTAATATATTTATTACCGATTAAGT
TTCAATGAACTAACTTGGATAAGATATGAATTATTGAATATATATCTCACATATATCTCTT
AACGTACATAGTACGTAGTGTTCACCTATTCAGTGACTGATCAAGAGATTATTATGGTC
AATCACCTTTAAATGTAAATTTAATGATAAAAAATATTATTTTAAATTAAATTATTTTATTTT
TAATCTTTAAATTTAAATTTAACGGTAATTAATCACATGACTGTCTCTTAACATA

Figure S6. The sequence of two fragments (FvePP2C6-F1 and FvePP2C6-F2) from *FvePP2C6* promoter and one fragment (FvePP2*C1*-F1) from *FvePP2C1* promoter, which were used for yeast one-hybrid assays.

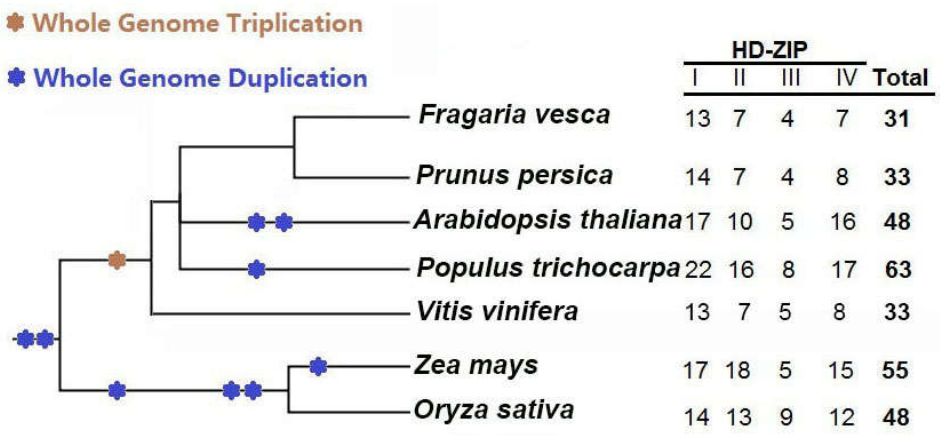

Figure S7. The Taxonomy Common Tree of the 7 species and the number of HD-Zip family genes in each species investigated. The information of HD-Zip family genes in different species (except *Fragaria vesca*) were obtained from previous studies [6, 7, 48, 49, 51]. The tree was constructed online by the Taxonomy Browser in NCBI (http://www.ncbi.nlm.nih.gov/Taxonomy/CommonTree/wwwcmt.cgi (accessed on 2 April 2022).
